# Phosphatidic Acid Stimulates Myoblast Proliferation through Interaction with LPA1 and LPA2 Receptors

**DOI:** 10.3390/ijms22031452

**Published:** 2021-02-01

**Authors:** Ana Gomez-Larrauri, Patricia Gangoiti, Natalia Presa, Asier Dominguez-Herrera, Chiara Donati, Paola Bruni, Miguel Trueba, Antonio Gomez-Muñoz, Alberto Ouro

**Affiliations:** 1 Department of Biochemistry and Molecular Biology, Faculty of Science and Technology, University of the Basque Country, P.O. Box 644, 48980 Bilbao (Bizkaia), Spain; anagola@hotmail.com (A.G.-L.); pgangoiti@hotmail.com (P.G.); npretor@gmail.com (N.P.); asier.dominguez@ehu.es (A.D.-H.); miguel.trueba@ehu.es (M.T.); 2 Respiratory Department, Cruces University Hospital, 48903 Barakaldo (Bizkaia), Spain; 3 Department of Experimental and Clinical Biomedical Sciences “Mario Serio”, University of Florence, Viale GB Morgagni 50, 50134 Firenze, Italy; chiara.donati@unifi.it (C.D.); paola.bruni@unifi.it (P.B.)

**Keywords:** phosphatidic acid, lysophosphatidic acid, myoblast proliferation, lysophosphatidic acid receptors

## Abstract

Phosphatidic acid (PA) is a bioactive phospholipid capable of regulating key biological functions, including neutrophil respiratory burst, chemotaxis, or cell growth and differentiation. However, the mechanisms whereby PA exerts these actions are not completely understood. In this work, we show that PA stimulates myoblast proliferation, as determined by measuring the incorporation of [^3^H]thymidine into DNA and by staining the cells with crystal violet. PA induced the rapid phosphorylation of Akt and ERK1/2, and pretreatment of the cells with specific small interferin RNA (siRNA) to silence the genes encoding these kinases, or with selective pharmacologic inhibitors, blocked PA-stimulated myoblast proliferation. The mitogenic effects of PA were abolished by the preincubation of the myoblasts with pertussis toxin, a Gi protein inhibitor, suggesting the implication of Gi protein-coupled receptors in this action. Although some of the effects of PA have been associated with its possible conversion to lysoPA (LPA), treatment of the myoblasts with PA for up to 60 min did not produce any significant amount of LPA in these cells. Of interest, pharmacological blockade of the LPA receptors 1 and 2, or specific siRNA to silence the genes encoding these receptors, abolished PA-stimulated myoblast proliferation. Moreover, PA was able to compete with LPA for binding to LPA receptors, suggesting that PA can act as a ligand of LPA receptors. It can be concluded that PA stimulates myoblast proliferation through interaction with LPA1 and LPA2 receptors and the subsequent activation of the PI3K/Akt and MEK/ERK1-2 pathways, independently of LPA formation.

## 1. Introduction

Phosphatidic acid (PA) is the precursor of triacylglycerol (TAG), which is the main form of energy store in humans, and constitutes the original building block from which many phospholipids are synthesized [1,2], thereby playing a relevant role in cell architecture. Later, it was demonstrated that PA is a second messenger capable of regulating a variety of intracellular signaling pathways and cell functions.

PA can be synthesized by three major pathways: (a) the acylation of lysophosphatidic acid (LPA, initially formed by the esterification of sn-1 by glycerol 3-phosphate acyltransferase) by LPA acyltransferases, (b) the phosphorylation of diacylglycerol (DAG) by DAG kinase, and (c) the hydrolysis of membrane phospholipids, namely phosphatidylcholine (PC), by phospholipase D (PLD) activities. Whilst LPA acyltransferases are involved in the biosynthesis of structural PA in the endoplasmic reticulum (ER), PA produced by DAG kinase or PLDs is implicated in the regulation of key physiological and pathological cell process. These include apoptosis, cytoskeletal rearrangement, chemotaxis, membrane trafficking, differentiation, micropinocytosis, secretory vesicle formation, cell growth, and tumor progression [3,4,5,6,7,8,9,10].

In mammalian cells, PA exerts its biological effects through distinct mechanisms. In this context, the cone-shaped geometry and negative charge of the molecule enable PA to interact with different proteins or enzymes to regulate their catalytic activities and their association with different membrane compartments. This is, for instance, the case for some GTP-binding proteins of the ADP ribosylation factor (Arf) and Rho families of homomeric G proteins [11]. Among the main signaling regulators, PA has been shown to control the activity of various protein kinase C (PKC) isoforms to promote the recruitment and activation of the proto-oncogene kinase Raf [12], and to regulate the activity of the mammalian target of rapamycin (mTOR), which are all involved in the regulation of cell proliferation [13]. In addition to kinases, PA also modulates the activity of a variety of phosphatases, including protein phosphatase 1 (PP1), which is involved in many cellular activities such as glycogen metabolism, the processing of RNA and the regulation of the cell cycle [10]. Although PA is a signaling molecule in its own right, some of its biological effects can be exerted by its conversion to LPA by phospholipase A_2_ activity [14]. Concerning muscle biology, the proliferation of myogenic cells is a critical process in skeletal muscle formation during embryogenesis, or the regeneration of adult muscle after injury. Muscle regeneration results from the activation, proliferation, and fusion of myogenic precursor cells called satellite cells. If left unrepaired, muscle damage can lead to loss of muscle mass, locomotive deficiency, and in severe cases, death. An important effector that leads to muscle regeneration is the mechanical stimulation. This plays a major role in the regulation of skeletal muscle mass and its maintenance contributes to prevent disease and to improve life quality. Some studies have shown that the mechanical stimulation of skeletal muscle and exercise promote an increase in PA concentration [15,16,17,18]. Additionally, PA is found in plasma and is present in a number of commonly consumed food sources [19]. However, the mechanisms by which PA regulates cell proliferation are not well understood.

In the present work, we demonstrate that PA stimulates myogenic cell proliferation through interaction with the LPA receptors, LPA1 and LPA2, and the subsequent activation of the phosphatidylinositol 3 kinase (PI3-K)/Akt (also known as protein kinase B) and extracellularly regulated kinases (ERK1/2) signaling pathways.

## 2. Results

### 2.1. Phosphatidic Acid Promotes Myoblast DNA Synthesis and Proliferation

**Figure 1:**
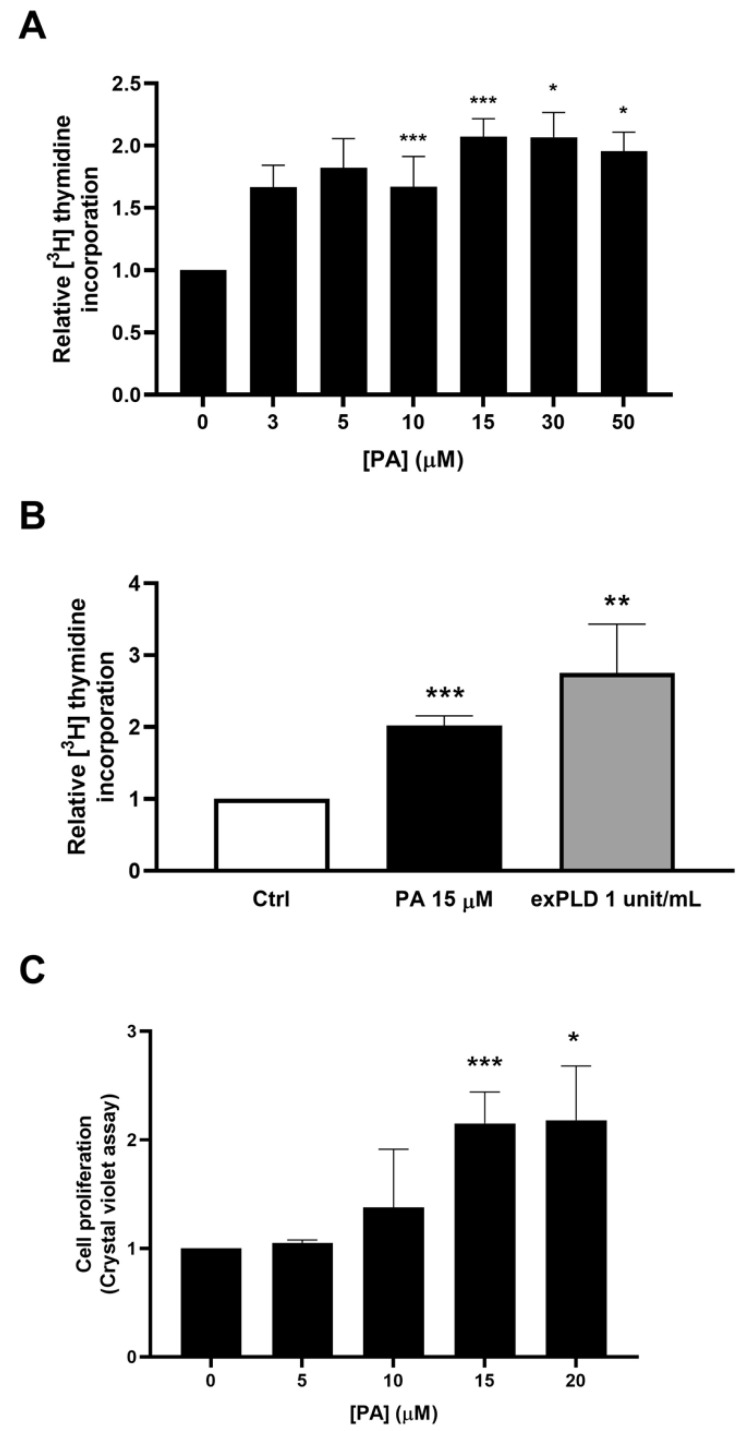
Phosphatidic acid (PA) stimulates C2C12 myoblast proliferation. Approximately 40% confluent C2C12 myoblasts were serum-starved for 24 h in Dulbecco’s modified eagle’s medium (DMEM) supplemented with 0.1% bovine serum albumin (BSA). (**A**) Cells were treated for 16 h with PA at the indicated concentrations. [^3^H]Thymidine incorporation into DNA was measured as described in the Materials and Methods section. Data are expressed relative to the control (Ctrl) value without agonist and are the means ± SEM of 3 independent experiments performed in triplicate. (*** *p* < 0.001). (**B**) Cells were treated for 16 h with 15 μM PA or with exogenous phospholipase D (exPLD) (1 unit/mL). [^3^H]Thymidine incorporation into DNA was measured as indicated in the Materials and Methods section. Data are expressed relative to the control value (Ctrl) without agonist and are the means ± SEM of 4 independent experiments performed in triplicate. (** *p* < 0.01; *** *p* < 0.001). (**C**) Cells were treated for 24 h with PA at the indicated concentrations. Cell proliferation was determined by staining the myoblasts with crystal violet as described in the Materials and Methods section. Data are expressed relative to the control value without agonist and are the means ± SEM of 6 independent experiments performed in triplicate. (* *p* < 0.05; *** *p* < 0.001).

In the present work, we show that PA stimulates DNA synthesis and cell division in C2C12 myoblasts, a murine satellite cell line commonly employed to investigate skeletal muscle cell biology. The stimulation of DNA synthesis was concentration dependent, reaching maximal values at 15 μM PA after 16 h of incubation (Figure 1A). Likewise, the treatment of C2C12 cells with exogenous phospholipase D (exPLD), which generates PA at the plasma membrane of cells, also stimulated DNA synthesis in the myoblasts (Figure 1B). Cell proliferation was also determined by staining the myoblasts with crystal violet, which is a reliable assay to estimate the cell number of adherent cells [20]. Treatment with PA for 16 h significantly increased cell proliferation, with optimal stimulation being attained at 15 µM PA (Figure 1C).

### 2.2. Phosphatidic Acid Stimulates the PI3-K/Akt and MEK/ERK1-2 Pathways in Myoblasts. Implication in PA-Stimulated Myoblast Proliferation

**Figure 2:**
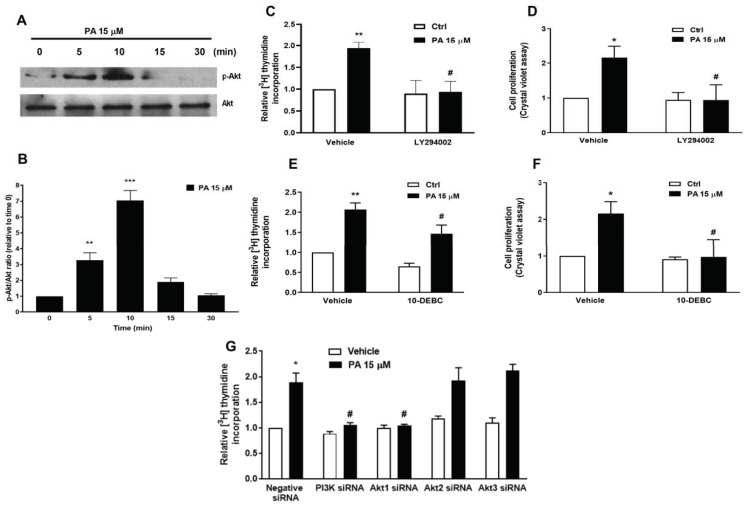
Involvement of the PI3-K/Akt pathway in PA-stimulated myoblast proliferation. Myoblasts were serum-starved for 24 h in DMEM supplemented with 0.1% BSA. (**A**) The cells were challenged with 15 μM PA at the indicated times. Cell lysates were analyzed by western blotting as described in the Materials and Methods section. Phosphorylation of Akt was determined with an antibody specific to phospho-Akt (Ser 473). Equal loading of protein was monitored using a specific antibody to total Akt. Panel A corresponds to a representative blot of three independent experiments. (**B**) Results of scanning densitometry of the exposed film showing the p-Akt/total Akt ratio. Data are expressed relative to the control value without agonist and are the mean ± SEM of 3 independent experiments performed in triplicate. (** *p* < 0.01; *** *p* < 0.001). (**C**) C2C12 myoblasts were preincubated for 30 min with 1 μM LY 294002 and then treated with 15 μM PA for 16 h. [^3^H]Thymidine incorporation was measured as described in the Materials and Methods section. Results are expressed relative to the control value without agonist and are the mean ± SEM of 3 independent experiments performed in triplicate. (** *p* < 0.01, control versus PA-treated cells, # *p* < 0.05; PA-treated cells versus PA-treated cells in the presence of the inhibitor). (**D**) Cells were treated as in panel C. Cell proliferation was determined by staining the myoblasts with crystal violet as described in the Materials and Methods section. Results are expressed relative to the control value without agonist and are the mean ± SEM of 3 independent experiments performed in triplicate. (* *p* < 0.05, control versus PA-treated cells, ^#^ *p* < 0.05; PA-treated cells versus PA-treated cells in the presence of the inhibitor). (**E**) C2C12 cells were preincubated for 30 min with 1 μM 10-DEBC and then treated with 15 μM PA for 16 h. [^3^H]Thymidine incorporation was measured as described in the Materials and Methods section. Results are expressed relative to the control value without agonist and are the mean ± SEM of 3 independent experiments performed in triplicates. (** *p* < 0.01, control versus PA-treated cells, # *p* < 0.05; PA-treated cells versus PA-treated cells in the presence of the inhibitor). (**F**) Cells were treated as in E. Cell proliferation was determined by staining the myoblasts with crystal violet as described in the Materials and Methods section. Results are expressed relative to the control value without agonist and are the mean ± SEM of 3 independent experiments performed in triplicate. (* *p* < 0.05, control versus PA-treated cells, # *p* < 0.05; PA-treated cells versus PA-treated cells in the presence of the inhibitor). (**G**) C2C12 cells were preincubated for 5 h with negative (scrambled) siRNA, or with specific PI3K, Akt1, Akt2, or Akt3 siRNA (40 nM in all cases), prior to stimulation with 15 μM PA, as indicated. [^3^H]Thymidine incorporation was measured as described in the Materials and Methods section. Results are expressed relative to the control value without agonist and are the mean ± SEM of 3 independent experiments performed in triplicate. (* *p* < 0.05 control versus PA-treated cells, # *p* < 0.05; PA-treated cells versus PA-treated cells in the presence of the corresponding siRNA).

To investigate the mechanisms by which PA exerts is mitogenic actions, we tested the effects of this phospholipid on the stimulation of PI3-K/Akt and MEK/ERK1-2, which are two major signaling pathways implicated in the regulation of cell proliferation by growth factors [21,22]. The treatment of C2C12 myoblasts with PA caused a rapid and time dependent phosphorylation of Akt (at Ser 473), which is a kinase downstream of PI3-K activation (Figure 2A,B). To test whether this pathway is involved in the mitogenic effect of PA, the myoblasts were preincubated with selective inhibitors of PI3-K or Akt and DNA synthesis was determined after stimulation with PA. Figure 2, panels C to F, shows that the selective PI3-K inhibitor LY294002, or the blockade of Akt activity with 10-DEBC, a specific inhibitor of this kinase, substantially reduced the incorporation of [^3^H]thymidine into DNA and the increase in cell number that were elicited by PA. Likewise, the preincubation of these cells with specific siRNAs to silence the genes encoding PI3-K or Akt1 completely blocked the stimulation of DNA synthesis by PA, suggesting that the PI3-K/Akt1 pathway is implicated in the mitogenic action of PA. Knockdown of Akt2 or Akt3 were without effect, indicating that Akt1 is the relevant isoform involved in this pathway (Figure 2G).

**Figure 3:**
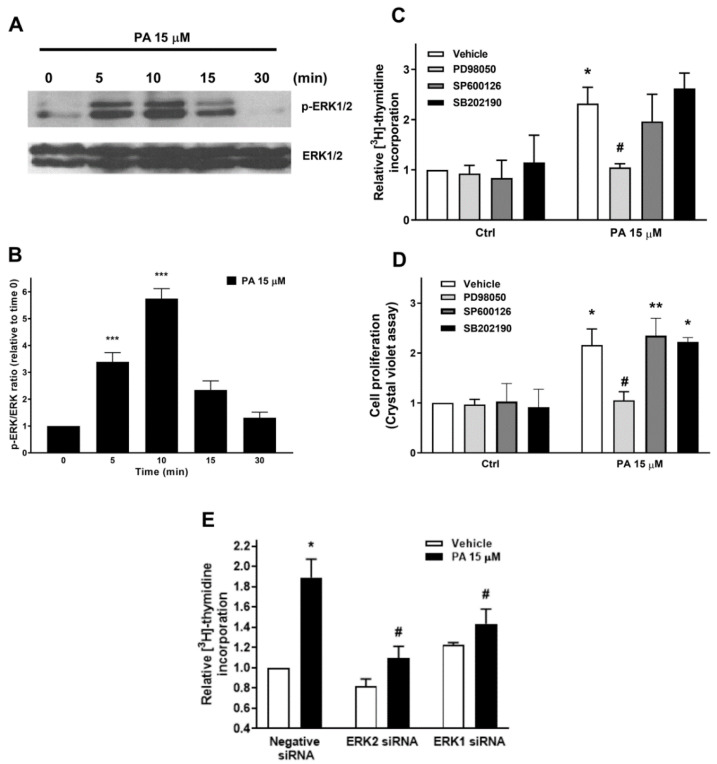
Involvement of MEK/ERK 1/2, but not p38 or JNK in PA-stimulated myoblast proliferation. Myoblasts were serum starved and maintained in DMEM supplemented with 0.1% BSA for 24 h before addition of agonists. (**A**) The cells were challenged with 15 μM PA at the indicated time points. Cell lysates were analyzed by western blotting as described in the Materials and Methods section. Phosphorylation of ERK1/2 was determined with an antibody specific to phospho-ERK1/2. Equal loading of protein was monitored using a specific antibody to total ERK1/2. Panel A shows a representative blot of three independent experiments. (**B**) Results of scanning densitometry of the exposed film showing the p-ERK/total ERK ratio. Data are expressed relative to the control value without agonist and are the mean ± SEM of 3 independent experiments performed in triplicate. (*** *p* < 0.001). (**C**) C2C12 cells were preincubated for 30 min with 10 μM PD98059 to inhibit MEK, 5 μM SB239063 to inhibit p38 or 1 μM SP600125 to inhibit JNK, and were then treated with 15 μM PA, or vehicle for 16 h. [^3^H]Thymidine incorporation was measured as described in the Materials and Methods section. Results are expressed relative to the control value without agonist and are the mean ± SEM of 3 independent experiments performed in triplicate. (* *p* < 0.05, control versus PA-treated cells, # *p* < 0.05; PA-treated cells versus PA-treated cells in the presence of the inhibitor). (**D**) Cells were treated as in panel C. Cell proliferation was determined by staining the myoblasts with crystal violet as described in the Materials and Methods section. Results are expressed relative to the control value without agonist and are the mean ± SEM of 3 independent experiments performed in triplicate. (* *p* < 0.05, control versus PA-treated cells, # *p* < 0.05; PA-treated cells versus PA-treated cells in the presence of the inhibitor); ** *p* < 0.01, control versus PA-treated cells in the presence of SP600126; *p* < 0.05, control versus PA-treated cells in the presence of SB202190. (**E**) C2C12 cells were pre-treated for 5 h with negative (scrambled) siRNA, or with specific ERK1 or ERK2 siRNA (40 nM in all cases), prior to stimulation with 15 μM PA, as indicated. [^3^H]Thymidine incorporation was measured as described in the Materials and Methods section. Results are expressed relative to the control value without agonist and are the mean ± SEM of 3 independent experiments performed in triplicate. (* *p* < 0.05, control versus PA-treated cells, # *p* < 0.05; PA-treated cells versus PA-treated cells in the presence of the corresponding siRNA).

We also observed that PA induced the rapid phosphorylation of ERK1-2, which are MAPKs downstream of MEK activation (Figure 3A,B). To evaluate the implication of this pathway in the mitogenic actions of PA, the selective MEK inhibitor PD98059 was used. Figure 3 (panels C and D) shows that both the stimulation of DNA synthesis and the increase in cell number were completely inhibited by PD98059. Moreover, the treatment of the myoblasts with specific siRNA to silence the genes encoding ERK1-2 completely blocked PA-stimulated DNA synthesis, suggesting that the MEK/ERK1-2 pathway is also relevant in this process. By contrast, the preincubation of the myoblasts with SB21091 or SP600125, which selectively inhibit the MAPKs p38 and JNK, respectively, did not significantly alter the mitogenic effect of PA in these cells (Figure 3C,D). Likewise, the preincubation of these cells with specific siRNAs to silence the genes encoding ERK1 or ERK2 completely blocked the stimulation of DNA synthesis by PA, suggesting that the MEK/ERK1-2 pathway is also implicated in the mitogenic action of PA (Figure 3E).

### 2.3. Phosphatidic Acid Stimulates Myoblast Proliferation Through Interaction with the LPA Receptors LPA1 and LPA2

**Figure 4:**
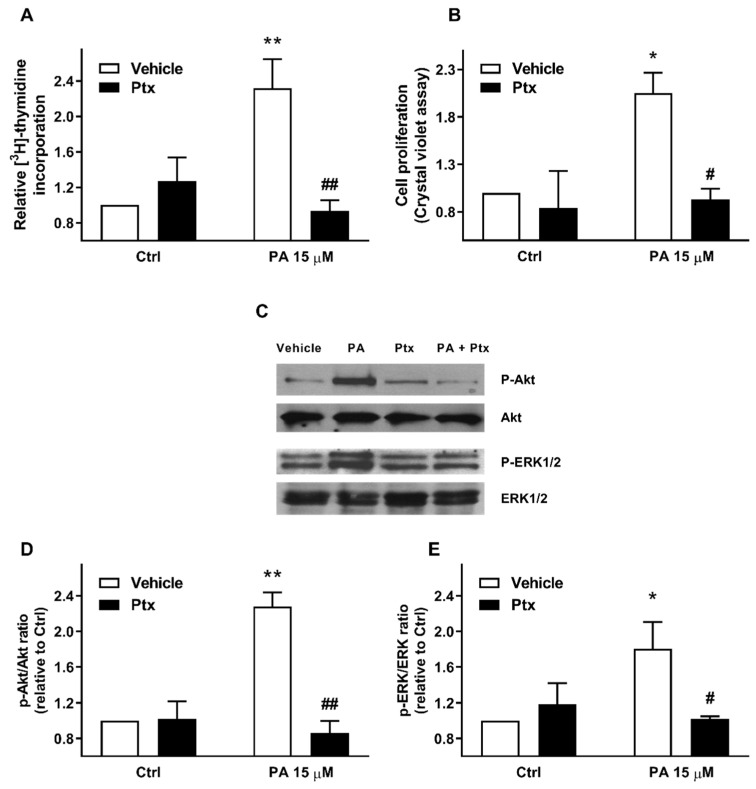
Pertussis toxin inhibits PA-stimulated myoblast proliferation and Akt and ERK1/2 phosphorylation. Myoblasts were serum-starved in DMEM supplemented with 0.1% BSA for 24 h. (**A**) Cells were preincubated for 16 h with 0.5 μg/mL pertussis toxin (Ptx) and then further treated with 15 μM PA or vehicle for 16 h, as indicated. [^3^H]Thymidine incorporation was measured as described in the Materials and Methods section. Results are expressed relative to the control value without agonist and are the mean ± SEM of 3 independent experiments performed in triplicate. (** *p* < 0.01, control versus PA-treated cells, ## *p* < 0.01; PA-treated cells versus PA-treated cells in the presence of Ptx). (**B**) Cells were treated as in panel A. Cell proliferation was determined by staining the myoblasts with crystal violet as described in the Materials and Methods section. Results are expressed relative to the control value without agonist and are the mean ± SEM of 3 independent experiments performed in triplicate. (* *p* < 0.05, control versus PA-treated cells, # *p* < 0.05; PA-treated cells versus PA-treated cells in the presence of Ptx). (**C**) C2C12 cells were preincubated for 16 h with 0.5 μg/mL Ptx. The myoblasts were then challenged with 15 μM PA or vehicle for 5 min, as indicated. Cell lysates were analyzed by western blotting as described in the Materials and Methods section. Phosphorylation of Akt (Ser473) and ERK were determined using specific antibodies to phospho-Akt (P-Akt), or phospho-ERK1/2 (P-ERK1/2). Equal loading of protein was monitored using specific antibodies to total Akt and ERK1/2. Panel C shows a representative blot of three independent experiments. (**D**,**E**) Results of scanning densitometry of the exposed films. Data are expressed as arbitrary units of intensity relative to the control values in the absence of agonist or Ptx (Ctrl) and are the mean ± SEM of 3 replicate experiments. (* *p* < 0.05; ** *p* < 0.01; control versus PA-treated cells, # *p* < 0.05, ## *p* < 0.01; PA-treated cells versus PA-treated cells in the presence of Ptx, as indicated).

Another relevant observation in this work was that the stimulation of myoblast proliferation was completely abolished by the pretreatment of the myoblasts with pertussis toxin (Ptx), an inhibitor of heterotrimeric GTP binding proteins of the Gi family of proteins (Figure 4A,B), suggesting the implication of Gi protein-coupled receptors in this process. Moreover, Ptx completely blocked the rapid phosphorylation of Akt and ERK1-2 induced by PA (Figure 4C–E), further indicating that the PI3-K/Akt and MEK/ERK1-2 pathways are relevant for the stimulation of myoblast proliferation by this phospholipid.

**Figure 5:**
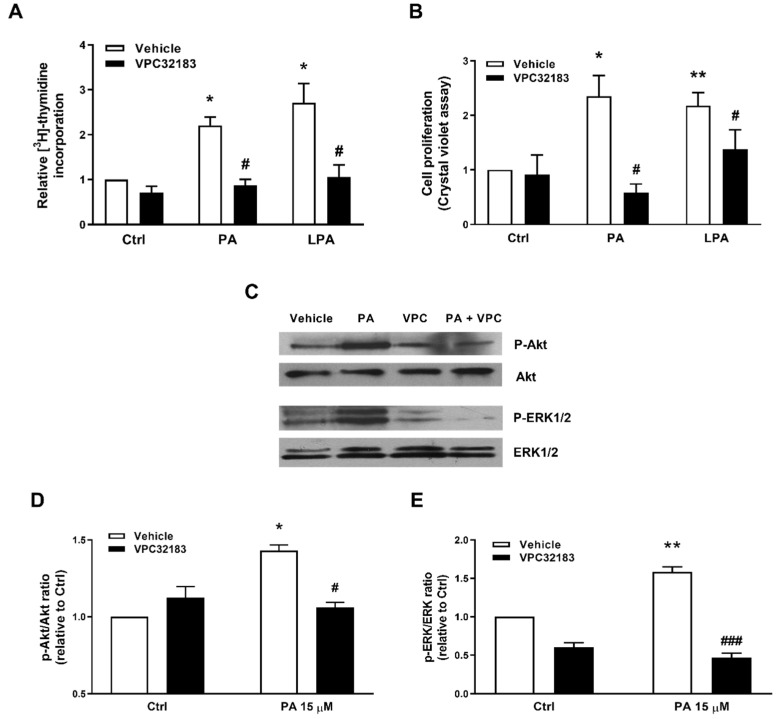
Inhibition of PA-stimulated myoblast proliferation and Akt and ERK1/2 phosphorylation by the lysoPA (LPA) receptor antagonist VPC32183. Myoblasts were serum-starved in DMEM supplemented with 0.1% BSA for 24 h. (**A**) The cells were preincubated for 30 min with 5 μM VPC32183 and were then challenged with 15 μM PA or 15 μM LPA for 16 h, as indicated. [^3^H]Thymidine incorporation was measured as described in the Materials and Methods section. Results are expressed relative to the control value without agonist and are the mean ± SEM of 3 independent experiments performed in triplicate. (* *p* < 0.05, control versus PA or LPA-treated cells, # *p* < 0.05; PA or LPA-treated cells versus PA or LPA-treated cells in the presence of VPC32183). (**B**) Cells were treated as in panel A. Cell proliferation was determined by staining the myoblasts with crystal violet as described in the Materials and Methods section. Results are expressed relative to the control value without agonist and are the mean ± SEM of 3 independent experiments performed. Control versus PA-treated cells; ** *p* < 0.01, control versus LPA-treated cells; # *p* < 0.05; PA- or LPA-treated cells versus PA- or LPA-treated cells in the presence of VPC32183). (**C**) Cells were preincubated with 5 μM VPC32183 for 30 min and then treated with 15 μM PA for 5 min. Cell lysates were analyzed by western blotting as described in the Materials and Methods section. Phosphorylation of Akt and ERK1/2 was determined using specific antibodies against phospho-Akt (P-Akt) or phospho-ERK1/2 (P-ERK1/2). Equal loading of protein was monitored using specific antibodies to total Akt and total ERK1/2. Panel C shows a representative blot of three independent experiments. (**D**,**E**) Results of scanning densitometry of the exposed films. Data are expressed as arbitrary units of intensity relative to the control siRNA values in the absence of agonist or inhibitor (Ctrl) and are the mean ± SEM of 3 replicate experiments. (* *p* < 0.05; ** *p* < 0.01; control versus PA-treated cells, # *p* < 0.05, ### *p* < 0.001; PA-treated cells versus PA-treated cells in the presence of VPC32183, as indicated).

These findings suggested that PA might be acting through interaction with a Gi protein-coupled receptor to elicit its mitogenic effects. Since a specific receptor for PA has not yet been identified, we hypothesized that PA might act through interaction with any of the LPA receptors that are expressed in the myoblasts. In this connection, RT-PCR studies revealed the presence of LPA receptor mRNAs in satellite cells [23] and in C2C12 myoblasts [24]. Of note, contrary to LPA1 and LPA2 receptors, the LPA4 receptor was implicated in a reduction in Akt and ERK1-2 phosphorylation rather than in their enhancement, and this receptor was coupled to Gq, G12/13 and Gs, but not to Gi proteins [25]. Hence, the intervention of LPA4 in PA-stimulated myoblast proliferation could be ruled out. To test the possible implication of LPA1 in this process, the selective inhibitor VPC32183 was used. This inhibitor was able to block both PA-stimulated DNA synthesis (Figure 5A) and cell proliferation (Figure 5B). Moreover, the phosphorylation of Akt and ERK1-2 was also inhibited by the VPC32183 inhibitor (Figure 5C–E).

**Figure 6:**
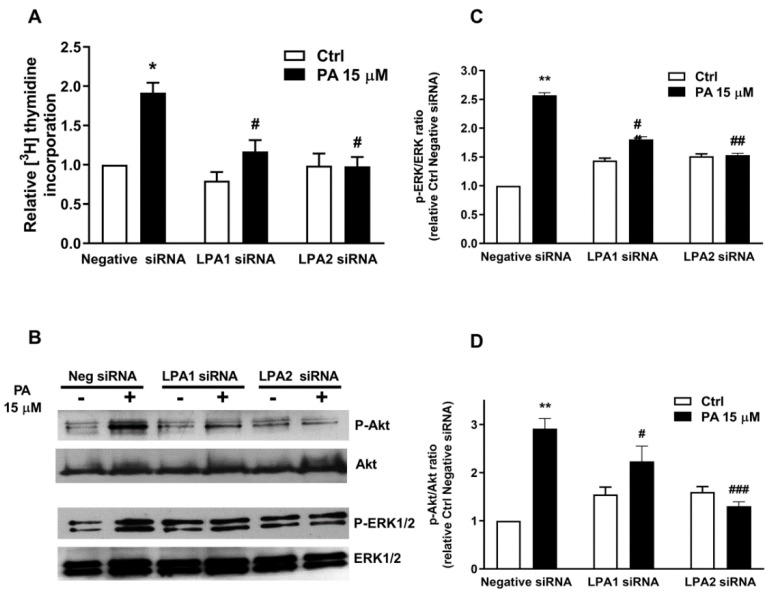
Implication of LPA1 and LPA2 receptors in PA-induced DNA synthesis and Akt and ERK phosphorylation. Myoblasts were preincubated for 5 h with negative (scrambled) siRNA (Neg siRNA) or with specific LPA1 or LPA2 siRNA (40 nM in all cases), as indicated. Transfected cells were maintained in DMEM with 10% fetal bovine serum (FBS) for 48 h and were then serum-starved in DMEM supplemented with 0.1% BSA for 24 h. (**A**) Cells were treated with 15 μM PA or vehicle for 16 h, as indicated. [^3^H]Thymidine incorporation was measured as described in the Materials and Methods section. Results are expressed relative to the control value without agonist and are the mean ± SEM of 3 independent experiments performed in triplicate. (* *p* < 0.05, control versus PA-treated cells, # *p* < 0.05; PA-treated cells versus PA-treated cells in the presence of siRNA). (**B**) Cells were treated as above and then stimulated for 5 min with 15 μM PA or vehicle as indicated. Cell lysates were analyzed by western blotting as described in the Materials and Methods section. Phosphorylation of Akt and ERK1/2 was determined using specific antibodies to phospho-Akt (P-Akt) or phospho-ERK1/2 (P-ERK1/2). Equal loading of protein was monitored using specific antibodies to total Akt or ERK1/2. Panel B shows a representative blot of three independent experiments. (**C**,**D**) Results of scanning densitometry of the exposed films. Data are expressed as arbitrary units of intensity relative to the control siRNA values in the absence of agonist (Ctrl) and are the mean ± SEM of 3 replicate experiments. (* *p* < 0.05; ** *p* < 0.01; control versus PA-treated cells, # *p* < 0.05, ## *p* < 0.01, ### *p* < 0.001; PA-treated cells versus PA-treated cells in the presence of the corresponding siRNA, as indicated).

In addition, specific siRNA to silence the gene encoding the LPA1 receptor abolished PA-stimulated DNA synthesis, and this was also the case when specific siRNA to silence LPA2 was used (Figure 6A). The silencing of the LPA1 and LPA2 receptors also blocked the phosphorylation of Akt and ERK1-2 (Figure 6B–D).

**Figure 7:**
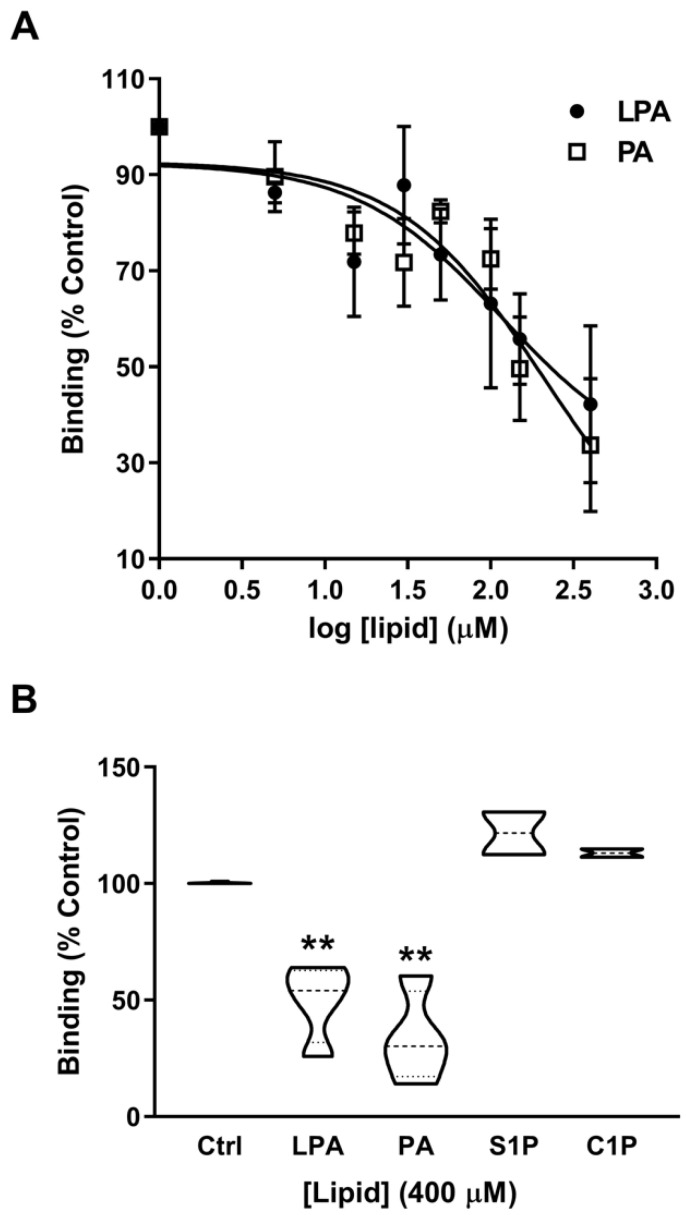
PA competes with LPA for binding to LPA receptors. (**A**) Myoblast membranes were incubated with 10 µM [^3^H]LPA in the presence of the indicated concentrations of unlabeled PA or LPA, as indicated. Non-specific binding was measured in the absence cell of membranes. Results are the mean ± SEM of three independent experiments performed in triplicate. Radioactivity of filter-bound radionuclide was quantified by liquid scintillation counting, as indicated in the Materials and Methods section. (**B**) The competition binding assays were performed with 10 µM [^3^H]LPA in the presence of 400 µM PA, 400 µM LPA, 400 µM S1P or 400 µM C1P, as indicated. Non-specific binding was measured in the absence of myoblast membranes. Results are the mean ± SEM of three independent experiments performed in triplicate. (** *p* < 0.01). Radioactivity of filter-bound radionuclide was quantified by liquid scintillation counting.

These findings suggest that both LPA1 and LPA2 receptors are implicated in the mitogenic actions of PA in myoblasts. To confirm that PA was able to bind to LPA receptors, radioligand binding assays using [^3^H]-labeled LPA and myoblast cell membranes were performed (see Materials and Methods). Figure 7A shows that there was competition for the specific binding of [^3^H]-labeled LPA by unlabeled LPA, whose IC_50_ (concentration of competing ligand that displaces 50% of the specific binding of the radioligand, [^3^H]-LPA) was 116.14 ± 3.14 µM. Of note, PA was able to also potently displace bound [^3^H]-LPA from myoblast membranes with an IC_50_ of 207.97 ± 2.21 µM. However, sphingosine-1-phosphate, a lysophosphosphingolipid that is structurally similar to LPA, and ceramide-1-phosphate, which is structurally similar to PA, were unable to displace bound [^3^H]-LPA from the myoblast membranes (Figure 7B).

### 2.4. PA Stimulates Myoblast Proliferation in the Absence of LPA Formation

**Figure 8:**
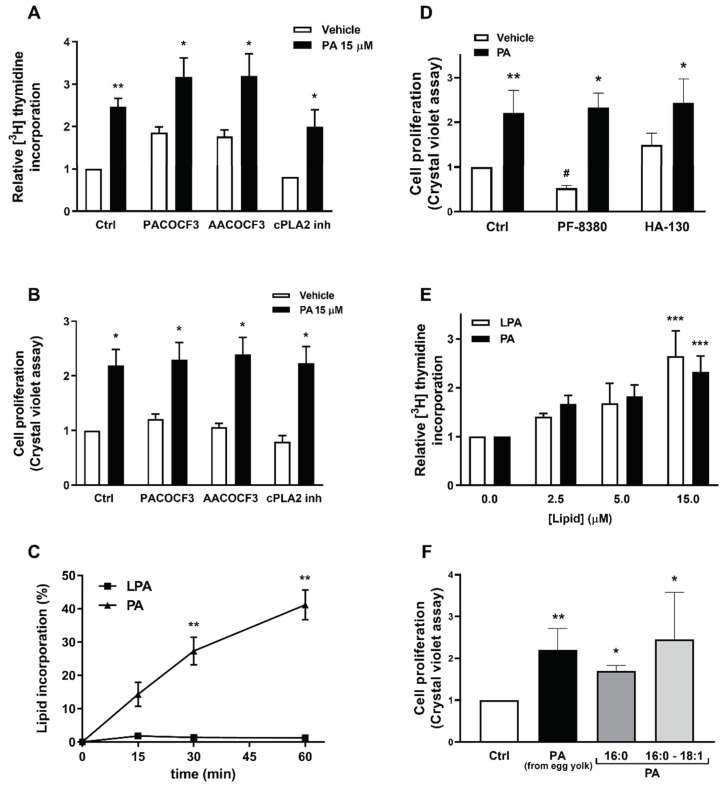
PA-stimulated myoblast proliferation does not depend on conversion of PA to LPA. Myoblasts were serum-starved in DMEM supplemented with 0.1% BSA for 24 h. (**A**) The cells were preincubated with 20 µM PACOCF3, 20 µM AACOCF3 or with 1 µM of the cPLA_2_ α inhibitor pyrrophenone (N-{(2S,4R)-4-(Biphenyl-2-ylmethyl-isobutyl-amino)-1-[2-(2,4-difluorobenzoyl)-benzoyl]-pyrrolidin-2-ylmethyl}-3-[4-(2,4-dioxothiazolidin-5-ylidenemethyl)-phenyl] acrylamide) for 30 min before they were challenged with 15 μM PA for 16 h. [^3^H]Thymidine incorporation into DNA was measured as described in the Materials and Methods section. Results are expressed relative to the control value without agonist and are the mean ± SEM of 3 independent experiments performed in triplicate (** *p* < 0.01, control versus PA-treated cells; * *p* < 0.05, control versus PA-treated cells in the presence of the indicated inhibitors. (**B**) Cells were treated as in panel A. Cell proliferation was determined by staining the myoblasts with crystal violet as described in the Materials and Methods section. Results are expressed relative to the control value without agonist and are the mean ± SEM of 3 independent experiments performed in triplicate. (* *p* < 0.05, control versus PA-treated cells in the absence or in the presence of the indicated inhibitors). (**C**) Cells were labelled with 15 μM [^14^C]PA (0.05 μCi/mL) for the indicated time points. PA and LPA levels were determined as described in the Materials and Methods section. Results are expressed as percentage of the radioactivity present in the PA and LPA thin-layer chromatography (TLC) spots compared to that in total lipids and are the mean ± SEM of 3 independent experiments performed in duplicate. (**D**) The cells were preincubated with 300 nM HA-130 or 300 nM PF-8380 for 30 min before they were treated with 15 μM PA for 16 h. Cell proliferation was determined by staining the myoblasts with crystal violet as described in the Materials and Methods section. Results are expressed relative to the control value without agonist and are the mean ± SEM of 3 independent experiments performed in triplicate. (** *p* < 0.01, control versus PA-treated cells; * *p* < 0.05, control versus PA-treated cells in the presence of HA-130, or PF-8380 treated cells versus PA-treated cells in the presence of this inhibitor; # *p* < 0.05, control versus cells incubated with PF-8380). (**E**) Cells were treated with PA or LPA for 16 h at the indicated concentrations. [^3^H]Thymidine incorporation into DNA was measured as described in the Materials and Methods section. Data are expressed relative to the control value without agonist and are the means ± SEM of 3 independent experiments performed in triplicate (*** *p* < 0.001, control versus PA- or LPA-treated cells). (**F**) Cells treated with PA from egg yolk or with different PA species (at 15 µM), as indicated. Results are expressed relative to the control value without agonist and are the mean ± SEM of 6 independent experiments performed in triplicate. (** *p* < 0.01, control versus PA from egg yolk; * *p* < 0.05, control versus 16:0-PA or 16:0-18:1-PA, as indicated).

Some previous studies suggested that PA exerts its biological effects through conversion to LPA by cytosolic PLA_2_ (cPLA_2_) activity, or that the PA samples used in experiments might have been contaminated with LPA [26]. However, the preincubation of the myoblasts with arachidonyl trifluoromethyl ketone (AACOCF3), Palmityl trifluoromethyl ketone (PACOCF3) or with pyrrophenone, which selectively inhibit cPLA2 activities, neither blocked PA-stimulated DNA synthesis (Figure 8A) nor cell proliferation (Figure 8B). In addition, the incubation of the myoblasts with [^14^C] phosphatidic acid for up to 1 h showed no conversion of PA into LPA (Figure 8C), thereby ruling out a possible intervention of LPA in the rapid phosphorylation of Akt and ERK1-2 by PA, which takes place in a few minutes (Figure 2 and Figure 3). Moreover, analysis of the PA samples used in experiments by thin layer chromatography rendered no detectable amount of LPA, in agreement with the compound supplier, who warrants a purity of 98% of the commercial PA samples. Some experiments were also performed in the presence of specific inhibitors of autotaxin, which is the most important enzyme for LPA production and generates most of the extracellular LPA [27,28]. Autotaxin is a lysophospholipase D enzyme that would efficiently convert any possible lysophosphatidylcholine (LPC) that might be present in the PA samples into LPA. However, the autotaxin inhibitor HA-130 at 300 nM, which is a concentration over 10-fold higher than its IC_50_ (28 nM) and was shown to potently inhibit transendothelial migration of naïve T lymphocytes [29], did not significantly alter PA-stimulated myoblast proliferation (Figure 8D). Additionally, the autotaxin inhibitor PF-8380 (300 nM), which significantly reduced basal myoblast proliferation, did not alter the mitogenic effect of PA (Figure 8D). It should also be noted that the optimal concentrations of PA and LPA to stimulate DNA synthesis in the myoblasts are similar (Figure 8E), also suggesting that the effect of PA is independent of LPA formation. Lastly, myoblast proliferation was tested using synthetic PA that was provided by Avanti Polar Lipids, who warrants a purity of the PA preparations higher than 99%, and these preparations are not expected to contain any other lipid species. The two different species of synthetic PA used in the experiments, 1,2-dipalmitoyl-*sn*-glycerol-3-phosphate (16:0-PA) and 1-palmitoyl-2-oleoyl-*sn*-glycerol-3-phosphate (16:0-18:1-PA), stimulated myoblast proliferation to the same extent as the natural mixture of egg yolk PA (Figure 8F), again suggesting that the stimulation of myoblast proliferation by PA is not caused by contamination with LPA.

## 3. Discussion

Muscle development and regeneration after injury seem to follow the same procedures. These rely on the activation of different cell types, namely satellite cells, and the formation of new myofibers under the control of myogenic regulators, including mitogenic growth factors or cytokines that are released by inflammatory cells [30,31,32]. However, the exact functions and effects of these factors on muscle remodeling and how they have evolved are not well understood. In the present work, we demonstrate that PA, a glycerophospholipid with mitogenic properties, stimulates myoblast proliferation, and we have identified at least part of the mechanisms by which PA regulates this process. Specifically, the treatment of mouse myoblasts with PA, or with exogenous PLD to generate PA at the plasma membrane of cells, resulted in the stimulation of the MEK/ERK1-2 and PI3K/Akt pathways leading to myoblast proliferation. These findings are consistent with the PA myogenic stimulation of mTOR, which is downstream of Akt or ERK1-2, in different cell types or tissues, including skeletal muscle [13,18,33,34]. In a recent report, PA has also been demonstrated to stimulate cell proliferation and promote G1/S phase transition through the activation of mTOR in primary muscle cells of the fish turbot (*Scophthalmus maximus*) [35], which, given the disparate growth pattern compared to mammals, suggests a well conserved action of PA through evolution.

The rapid phosphorylation of Akt and ERK triggered by PA suggests that it may be a receptor-mediated effect. In this regard, there is evidence suggesting that some PA actions are elicited through interaction with G protein-coupled receptors [11,36,37,38,39]. Specifically, the PA-stimulated Akt and ERK phosphorylation followed by the upregulation of DNA synthesis and cell proliferation that we observed in this work were all inhibited by Ptx, suggesting the intervention of Gi protein-coupled receptors in the regulation of these processes. PA also stimulated actin polymerization within seconds, leading to human monocyte migration, an effect that was optimal after 90 min of PA addition to the cells and that was also inhibited by Ptx [40]. Although the concentrations of PA that stimulated myoblast proliferation (10–15 µM) are higher than the PA concentrations found in plasma (3 µM) [41], local PA concentrations may be increased under certain circumstances. In this regard, the intracellular concentrations of PA in some cell types such as cardiomyocytes are around 20 µM, and can be even higher in hepatocytes [42,43]; if these amounts of PA were to be released upon cell activation, local concentrations of extracellular PA in the range 10–15 µM would likely be achievable in vivo. In this connection, it has been shown that 20 µM PA increases intracellular Ca^2+^ concentration through direct interaction with LPA receptors [44], an action that is compatible with the stimulation of cell proliferation by PA. Additionally, PA and phospholipase D2 (PLD2), a major enzyme that produces PA at the plasma membrane of cells, are enriched in extracellular vesicles [45,46], which can be secreted by most, if not all, cell types and bind to plasma membrane receptors to regulate cell responses [47]. Moreover, the release or accumulation of these vesicles at specific extracellular locations might increase extracellular PA concentrations locally.

Another key finding in the present study was that PA elicited its mitogenic effects in the myoblasts through interaction with the LPA1 and LPA2 receptors in the absence of LPA formation. In this connection, another study showed that the inhibition of LPA1/LPA3 receptors blocked PA induced morphological changes in C6 glioma cells [48]. In addition, PA stimulated the migration of human neutrophils and human leukemia HL-60 monocytes, and these actions could not be recapitulated by LPA [49]. Interestingly, LPA receptors mediated the mitogenic action exerted by the bioactive sphingolipid ceramide 1-phosphate through the stimulation of the LPA signaling axis [50]. In this work, a broader versatility of these receptors in relation to receptor-ligand interaction is provided, being activated also by PA. Moreover, as previously demonstrated in skeletal muscle precursor cells [26,51], PA stimulated DNA synthesis with similar potency to that of LPA when administered at the same concentrations, indicating that a possible contamination of the PA preparations with LPA cannot account for the mitogenic effect of PA (if PA were to be contaminated by LPA, 2% of the optimal concentration of PA used in this work (15 µM) would translate into a concentration equivalent to 0.3 µM LPA, which is not sufficient to stimulate myoblast proliferation). Moreover, the inhibition of the two major enzymes for LPA formation, PLA_2_ acting on PA or autotaxin acting on LPC, did not alter PA-stimulated cell proliferation, and pure synthetic 16:0-PA and 16:0-18:1-PA also potently stimulated myoblast proliferation. Additionally, the stimulation of human monocyte migration by suboptimal concentrations of PA was 2-fold higher than similar concentrations of LPA [40], further suggesting that at least some PA actions are independent of conversion to LPA. There are at least two molecular models that can explain the possible interaction of PA with LPA receptors. First, Alderton and co-workers suggested that both PA and LPA may bind to the same receptors, with the additional acyl group in PA binding to a second site, through a hydrophobic interaction. The ecto-site binding would effectively clamp PA to the receptor, causing its activation. This site may be on the receptor itself or in the lipid milieu surrounding the receptor [52]. A second model would implicate the allosteric activation of the G protein-coupled LPA receptors by PA. In fact, G protein-coupled receptors (GPCRs) have been referred to as “allosteric machines” since they carry multiple, spatially distinct, yet conformationally linked ligand-binding sites [53].

In addition to stimulating myoblast proliferation, PA also promotes muscle cell differentiation [54,55,56]. Both cell proliferation and differentiation seem to be regulated by mTOR, although through different mechanisms. In fact, mTORC1 can also be activated by intracellular PA, independently of receptor interaction. Whilst PA-stimulated muscle cell proliferation requires the upregulation of the kinase activity of mTORC1 [54,57], PA-induced muscle cell differentiation is regulated by a kinase independent activity of mTOR, probably involving the activation of Igf2 muscle specific enhancer gene [56].

In cell growth regulation, the amino acid-sensing Vps34-PLD1 pathway leads to the direct activation of the mTORC1 kinase by PA [57,58], but the myogenic IGF-II pathway is independent of mTOR kinase activity [56]. During myogenesis, myoblasts must exit the cell cycle and undergo an ordered set of myogenic events. So, it may be speculated that PA would first stimulate myoblast proliferation events that will be followed by the induction of muscle cell differentiation into myocytes to promote myogenesis. These facts are in agreement with previous work showing that PLD1- and PLD2-derived PA have different, and at times opposed, effects in cell biology. In particular, whilst PLD1 deficiency impaired FcεRI-mediated mast cell degranulation, PLD2 deficiency enhanced it [59]. Additionally, PLD1 deficiency impaired F-actin disassembly, whereas PLD2 enhanced microtubule formation [59].

It can be concluded that PA stimulates myoblast proliferation through interaction with LPA1 and LPA2 receptors and the subsequent activation of the PI3K/Akt and MEK/ERK1-2 pathways, independently of LPA formation. These findings place PA as a key regulator of muscle regeneration and repair and may have a positive impact in ensuring healthy musculature.

## 4. Materials and Methods

### 4.1. Materials

Phosphatidic acid (from egg yolk lecithin), lysophosphatidic acid, pertussis toxin, crystal violet, SB21091, SP600125, LY294002, and PF-8380 (4-[3-(2,3-Dihydro-2-oxo-6-benzoxazolyl)-3-oxopropyl]-1-piperazinecarboxylic acid (3,5-dichlorophenyl)methyl ester were from Sigma-Aldrich (St Louis, MO, USA). Mouse skeletal muscle C2C12 cells were obtained from the American Type Culture Collection (Manassas, VA, USA). Dulbecco’s modified eagle’s medium (DMEM) was from Lonza. Fetal bovine serum (FBS), oligofectamine and OptiMEM medium were supplied by Gibco (Thermo Fisher Scientific) (Waltham, MA, USA). [Methyl-^3^H] thymidine, L-α-dipalmitoyl, phosphatidic acid, -[glycerol-^14^C(U)]-, and [oleoyl-9,10-^3^H]-lysophosphatidic acid were from Perkin Elmer (Boston, MA, USA). Goat anti-rabbit IgG horseradish peroxidase secondary antibody, and those to phospho-ERK1/2 (Thr-202/ Tyr-204), phospho-Akt (Ser 473), total Akt, and total ERK1/2 were purchased from Cell Signalling Technology (Beverly, MA, USA). The 10-DEBC hydrochloride and PLA_2_ inhibitors AACOCF3 and PACOCF3 were supplied by Tocris Biosciences (Bristol, UK). HA-130 (B-[3-[[4-[[3-[(4-Fluorophenyl)methyl]-2,4-dioxo-5-thiazolidinylidene]methyl]phenoxy]methyl]-phenyl]-boronic acid was from Echelon Biosciences Inc (Salt Lake City, UT, USA). Antibodies and small RNA interferences (siRNA) to LPA1-2 receptors, and the negative control siRNA, were from Santa Cruz Biotechnology (Dallas, TX, USA). Small RNA interferences (siRNA) to PI3-K, Akt and ERK1/2 were purchased from Ambion (Austin, TX, USA). PD98059 and the cPLA_2_ α inhibitor pyrrophenone (N-{(2S,4R)-4-(Biphenyl-2-ylmethyl-isobutyl-amino)-1-[2-(2,4-difluorobenzoyl)-benzoyl]-pyrrolidin-2-ylmethyl}-3-[4-(2,4-dioxothiazolidin-5-ylidenemethyl)-phenyl] acrylamide) were from Calbiochem (San Diego, CA, USA). Sphingosine-1-phosphate, N-palmitoyl-ceramide-1-phosphate (C1P), 1,2-dipalmitoyl-*sn*-glycerol-3-phosphate (16:0-PA), 1-palmitoyl-2-oleoyl-*sn*-glycerol-3-phosphate (16:0-18:1-PA) and the VPC32183 inhibitor were supplied by Avanti Polar Lipids (Alabaster, AL, USA).

### 4.2. Cell Culture

C2C12 myoblasts (obtained from the American Type Culture Collection, ATCC CRL-1772) were routinely grown in DMEM supplemented with 10% fetal bovine serum (FBS), 2 mM L-glutamine, 100 U/mL penicillin, and 100 μg/mL streptomycin in T75 tissue culture flasks at 37 °C in an atmosphere of 5% CO_2_. The culture medium was replaced every two days. The cells were passaged when about 70% confluent, taking care not to surpass 20 passages, and were used in experiments when approximately 40–50% confluent. The cells were serum-starved for 24 h in DMEM containing 1 mg/mL BSA prior to the addition of agonists or inhibitors. PA was added to cells, sonicated in water so as to avoid the use of organic solvents. When inhibitors were used, these were added to the cells 30 min prior to agonist addition.

### 4.3. Determination of DNA Synthesis

C2C12 cells were seeded and grown in 12-well plates at 20,000 cells/well, as indicated above. Before experiments, the myoblasts were serum-starved for 24 h in DMEM supplemented with 0.1% BSA, and were then incubated in the presence or absence of agonists for 16 h. [^3^H]Thymidine incorporation into DNA was measured as described previously [60]. Briefly, [^3^H]thymidine (0.5 μCi/mL) was added for the last 2 h of incubation. The medium was then removed and cells were washed twice with phosphate-buffered saline (PBS). Cells were washed twice with ice-cold PBS before the addition of 500 μL of 10% trichloroacetic acid for 5 min at 4 °C. The cells were washed again in ice-cold PBS, and 250 μL of ethanol:ether (3:1 *v/v*) was added to the insoluble material. Samples were then lysed in 0.25 M NaOH with 1% sodium dodecyl sulfate (SDS) for 1 h at 37 °C. Radioactivity was measured by scintillation counting.

### 4.4. Determination of Cell Proliferation

C2C12 myoblasts were seeded at 4000 cells/well in 96-well plates, and were grown and treated as indicated above. Before experiments, the myoblasts were serum-starved for 24 h in DMEM supplemented with 0.1% BSA, and were then incubated in the presence or absence of agonists for 24 h. Cell proliferation was estimated by staining the cells with crystal violet, as previously reported [61]. The crystal violet assay is a quick and reliable screening method that is suitable to estimate cell number for the examination of cell growth and survival [20,61,62]. Briefly, C2C12 myoblasts were stained with a solution of 0.5% crystal violet in 20% methanol for 20 min at 37 °C. The cells were then washed three time with PBS and left to dry. The dye was eluted by adding 200 µL of methanol to each well with shaking for 20 min, and the absorbance was read at 570 nm using a Power Wave XS plate reader from Biotek.

### 4.5. Small Interfering RNA (siRNA)-Mediated Depletion

C2C12 cells in OptiMEM were transfected with LPA1, LPA2, Akt, PI3-K, ERK1/2 or negative vector siRNA (40 nM) for 5 h using oligofectamine according to the manufacturer’s instructions. The transfected cells were maintained in DMEM with 10% FBS for 48 h before starting the experiments. In parallel, cell lysates were immunoblotted with specific antibodies to evaluate the efficiency of siRNA-mediated protein depletion.

### 4.6. Western Blot Analysis

C2C12 myoblasts were collected and lysed in ice-cold homogenization buffer as previously described [63]. Protein aliquots (20–40 μg) from each sample were loaded onto 12% polyacrylamide gels and separated by SDS-PAGE. Proteins were transferred to nitrocellulose membranes and were blocked for 1 h with 5% skim milk in Tris-buffered saline (TBS) containing 0.01% sodium azide (NaN_3_) and 0.1% Tween 20 to prevent non-specific antibody binding. The nitrocellulose membranes were then incubated overnight at 4 °C with the primary antibody dissolved in TBS/0.1% Tween. The nitrocellulose membranes were then washed three times with TBS/0.1% Tween 20, and were incubated with horseradish peroxidase-conjugated secondary antibody at 1:5000 dilution for 1 h. Protein bands were visualized using the enhanced chemiluminescence assay kit, Supersignal West Femto (from Pierce Biotechnology, Inc.).

### 4.7. Phosphatidic Acid Uptake and Metabolism

PA uptake by the myoblasts was studied by labelling C2C12 cells with 0.05 μCi/mL (111,000 dpm) of [^14^C]PA at 15 μM for the indicated times. The radioactive medium was then removed and the cells were washed twice with ice-cold calcium-free phosphate-buffered saline (PBS). They were then scraped into 0.5 mL of methanol. The plates were washed further with 0.5 mL of methanol, and the two methanol samples were combined and mixed with 0.5 mL of chloroform. Lipids were extracted by separation of phases with 0.5 mL of chloroform and 0.9 mL of a solution containing 2 M KCl in 0.2 M H_3_PO_4_. Chloroform phases were dried down under a stream of nitrogen and lipids were separated by thin-layer chromatography (TLC) using silica gel 60-coated plates. TLC plates were soaked in 2.3% boric acid in ethanol and developed with chloroform/ethanol/water/triethylamine (30:35:8:35, *v/v/v/v*) and then dried. Positions of PA and LPA were identified after staining with sulfuric acid in methanol by comparison with authentic standards. The radioactivity was quantified by liquid scintillation counting after scraping the corresponding spots from the TLC plates.

### 4.8. Preparation of Cell Membrane and LPA Radioligand-Binding Assay

The preparation of myoblast membranes and the LPA radioligand assay protocols have been previously described [64]. Briefly, myoblasts were incubated in homogenization buffer containing 10 mM Tris-HCl, 3 mM 2,2′,2″,2‴-(Ethane-1,2-diyldinitrilo)tetraacetic acid (EDTA), 3 mM Ethylene glycol-bis(2-aminoethylether)-N,N,N′,N′-tetraacetic acid (EGTA), 1mM NaF, protease inhibitors (10 µg/mL leupeptin, 10 µg/mL aprotinin, and 1 mM phenylmethylsulfonyl fluoride) for 30 min at pH 7.5 on ice. The myoblasts were lysed using a Dounce homogenizer and were centrifuged at 500× *g* for 5 min to remove unbroken cells and intact nuclei. Cell homogenates were then pelleted by centrifugation at 100,000× *g* for 30 min. The pellets were resuspended in a binding buffer containing 50 mM Tris-HCl, 150 mM NaCl, 0.8% fatty acid-free bovine serum albumin (BSA), 10 µg/mL leupeptin, 10 µg/mL aprotinin, and 0.2 mM phenylmethylsulfonyl fluoride, at pH 7.5. Only freshly prepared membranes were used in experiments. [^3^H]LPA (22,000 dpm/assay tube, at a final specific activity of 0.06 µCi/ pmol) and other lipids were sonicated in fatty acid-free BSA binding buffer and mixed with the cell membrane samples in a volume of 150 µL. The binding assays were performed in borosilicate tubes at 37 °C for 30 min with gentle mixing. The reactions were terminated by collecting the membranes onto glass monofiber filters (GF/C with a 1225 Sampling Manifold from Millipore. The filters were rapidly washed three times with 350 µL of ice-cold washing buffer containing 10 mM Tris-HCl and 15 mM NaCl, at pH 7.5. Radioactivity of filter-bound radionuclide was quantified by liquid scintillation counting.

### 4.9. Statistical Analysis

Results are expressed as means ± SEM of three independent experiments performed in triplicate, unless indicated otherwise. Statistical analyses were performed using one-way analysis of variance (ANOVA), or Student’s t-test as appropriate, with the level of significance set at *p* < 0.05.
